# Supplementary material for: Comprehensive Analysis of Seasonal and Geographical Variation in UVB Radiation Relevant for Vitamin D Production in Europe
Source: Nutrients. 2022 Dec 6;14(23):5189. doi: 10.3390/nu14235189 (PMC9735494; doi:10.3390/nu14235189)
Supplement: Supplementary file 1 [file nutrients-14-05189-s001.zip › nutrients-2040637-supplementary.pdf]

nutrients

MDPI

Supplementary Materials

Comprehensive Analysis of Seasonal and Geographical Variation in UVB Radiation Relevant for Vitamin D Production in Europe

Tarinee Khanna [1,†], Rasha Shraim [1,2,†], Masa Zarkovic [1,3], Michiel van Weele [4], Jos van Geffen [4] and Lina Zgaga [1,*]

Table S1. Capital Cities in Europe and their latitude and longitude, sorted according to their latitude. Negative Longitude numbers represent West (e.g. −5° E= 5° W).

City	Country	Longitude	Latitude
Nicosia	Cyprus	33.38	35.19
Valletta	Malta	14.51	36.90
Athens	Greece	23.73	37.98
Lisbon	Portugal	-9.15	38.77
Ankara	Turkey	32.88	39.95
Madrid	Spain	-3.70	40.42
Tirana	Albania	19.82	41.33
Rome	Italy	12.52	41.90
Skopje	Macedonia	21.43	42.00
Podgorica	Montenegro	19.26	42.43
Andorra la Vella	Andorra	1.52	42.51
Pristina	Kosovo	21.17	42.67
Sofia	Bulgaria	23.32	42.70
Monaco	Monaco	7.42	43.74
Sarajevo	Bosnia & Herzegovina	18.41	43.86
San Marino	San Marino	12.46	43.94
Bucharest	Romania	26.10	44.43
Belgrade	Serbia	20.45	44.79
Zagreb	Croatia	15.98	45.82
Ljubljana	Slovenia	14.51	46.06
Bern	Switzerland	7.45	46.95
Chisinau	Moldova	28.86	47.01
Vaduz	Liechtenstein	9.52	47.14
Budapest	Hungary	19.04	47.50
Bratislava	Slovakia	17.11	48.15
Vienna	Austria	16.35	48.23
Paris	France	2.34	48.85
Luxembourg	Luxembourg	6.13	49.61
Prague	Czech Republic	14.44	50.08
Kiev	Ukraine	30.52	50.45
Brussels	Belgium	4.36	50.80
London	Great Britain	-0.12	51.50
Warsaw	Poland	21.01	52.23
Amsterdam	Netherlands	4.90	52.37
Berlin	Germany	13.41	52.52
Dublin	Ireland	-6.25	53.33
Minsk	Belarus	27.56	53.90

Vilnius	Lithuania	25.28	54.69
Copenhagen	Denmark	12.57	55.68
Moscow	Russia	37.50	55.70
Riga	Latvia	24.11	56.95
Stockholm	Sweden	18.08	59.33
Tallinn	Estonia	24.75	59.44
Oslo	Norway	10.72	59.94
Helsinki	Finland	24.94	60.17
Reykjavik	Iceland	-21.82	64.13

Table S2. Summary of the retrieved studies reporting monthly 25(OH)D.

Study ID	Group	Reference	Participants
Denmark	healthy	Brot et al. 2001	2,016 healthy Danish perimenopausal women aged 45-58
EPIC	healthy	Roddan et al. 2007	730 incident fracture cases and 1,445 matched controls from the EPIC-Oxford study (mean age: 51 (men) and 53 (women))
FaroeIslands	healthy	Dalgard et al. 2010	713 elderly Faroese aged 70–74 years
Ireland	healthy	O'Neill et al. 2016	Irish adults (aged 18–84 years) from the nationally representative study
Sweden	healthy	Michaelsson et al. 2017	5,002 older women (mean age: 68 years) randomly selected from a large population-based cohort study
Sweden 2	healthy	Klingberg et al. 2015	healthy Swedish adult population; 540 blood donors (60% men; mean age: 41±13 years) and 75 thrombocyte donors (92% men, aged 46±11 years)
Switzerland	healthy	Guessour et al. 2012	1,309 subjects from a population-based study (age: 48.8±18.2, 48.7% male)
UK2	healthy	Webb et al. 2010	125 white adults aged 20–60
UKBB	healthy	UK Biobank dataset	443,842 participants (age: 57±8; 46.4% male)
Germany	diseased	Noe et al. 2017	625 HIV-infected patients (81% male, median age: 47 years)
Greece	diseased	Papadakis et al. 2015	population-based cohort of post-menopausal women with osteoporosis (mean age: 65.3 years)
Italy	diseased	Calgani et al. 2016	95 male urologic patients (mean age: 66 years; dominant conditions: benign prostate hypertrophy and prostate carcinoma)
Cyprus	lab	Xyda et al. 2022	data from 8780 Greek and 2594 Cypriot individuals (January 2013-October 2017) who had their 25(OH)D measured in two laboratories
Greece 2	lab		
Romania	lab	Niculescu et al. 2017	8,024 measurements from endocrinology center database (2012-2016), in individuals without a diagnosis of low bone mass
Turkey	lab	Solak et al.	the data of 39,107 patients who were admitted to hospital for any reason (January 1, 2016 to December 31, 2016) and whose serum 25(OH)D conc. was measured
Ukraine (A and Ch)	lab	Shchubelka 2020	1,639 adult and 184 children, randomly selected among those whose serum concentration of 25(OH) D was recorded in 2019 at the medical laboratory center

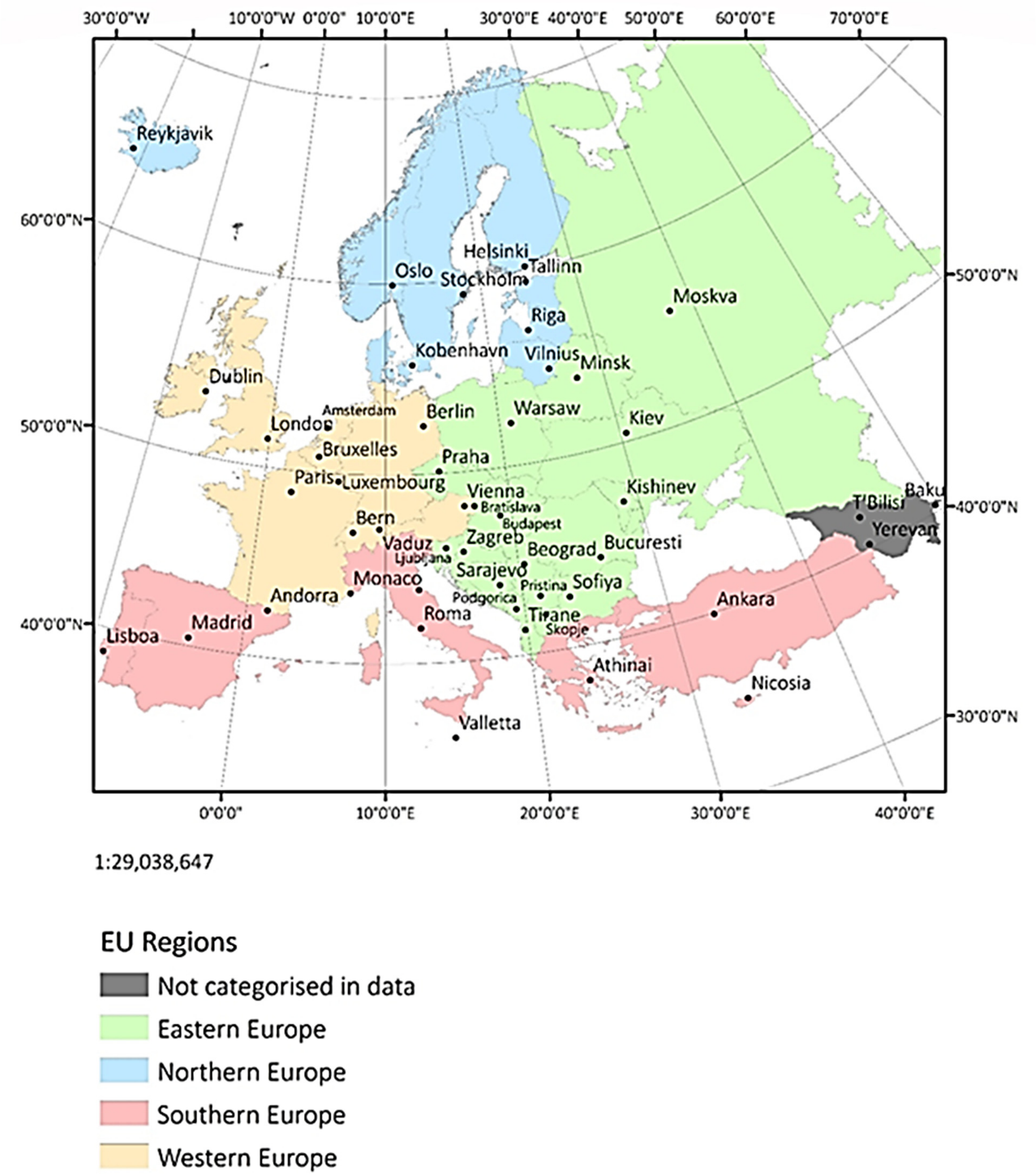

Figure S1. European countries and their capitals included in this study.

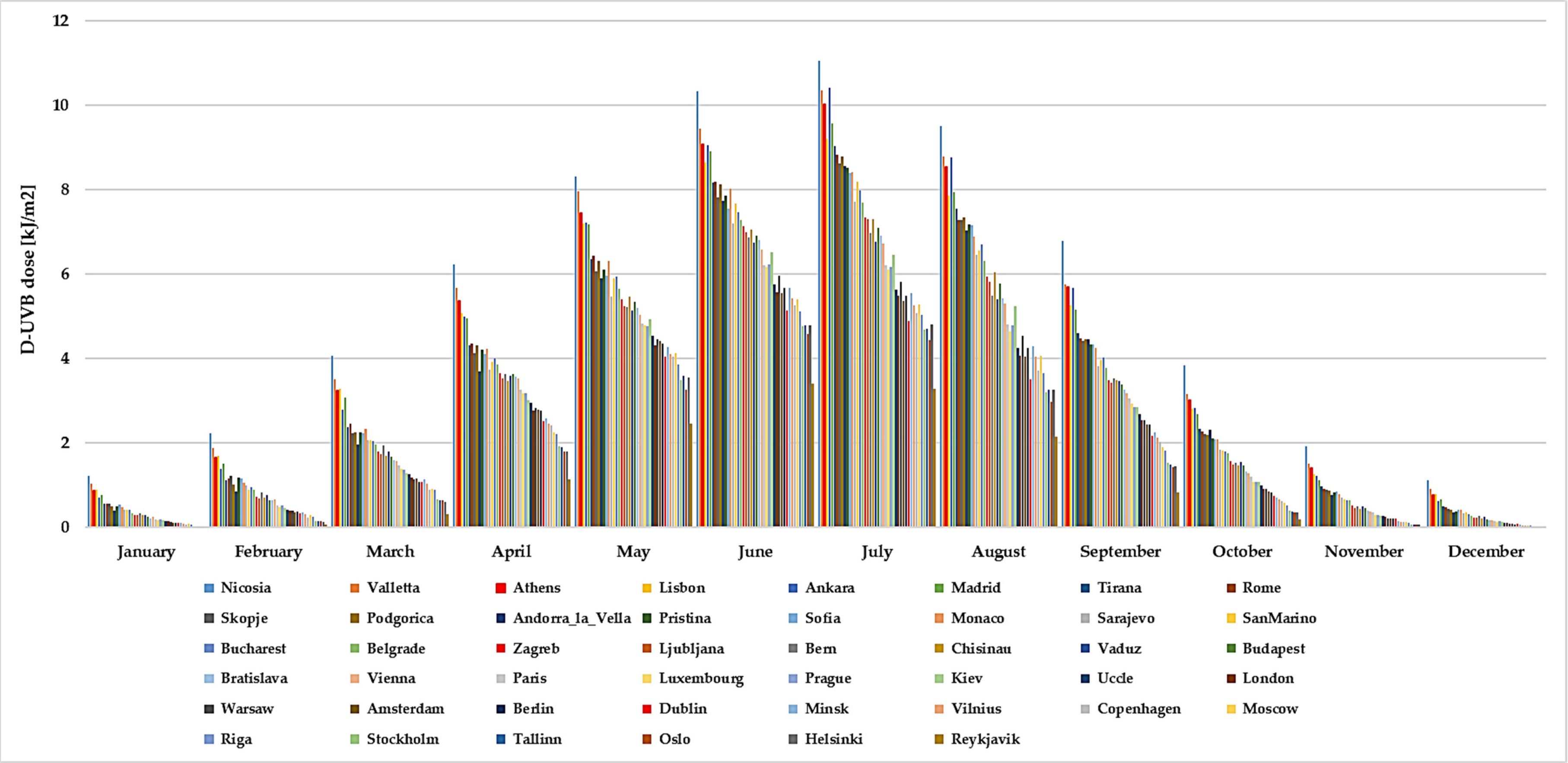

Figure S2. Mean daily D-UVB in European capital cities for each month of the year.

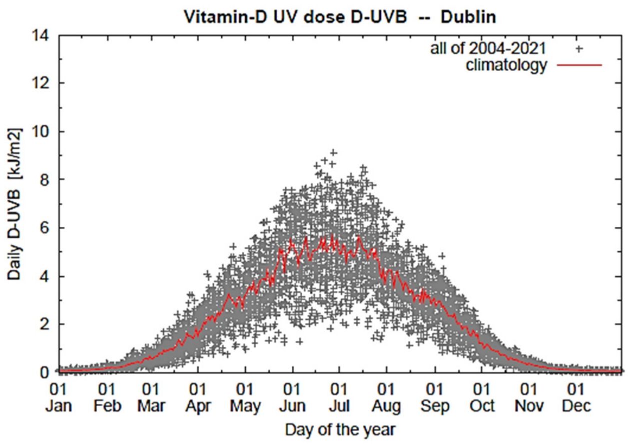

(A)

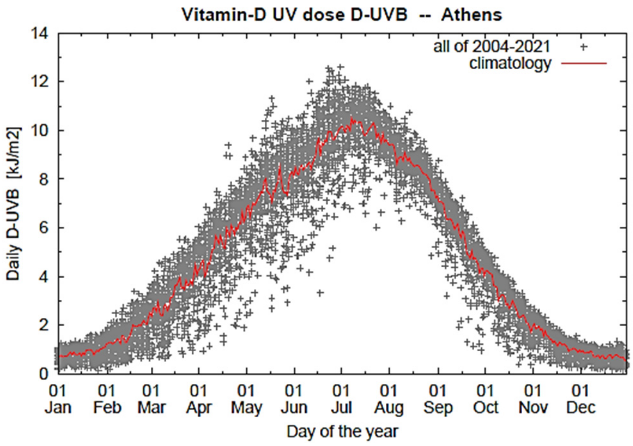

(B)

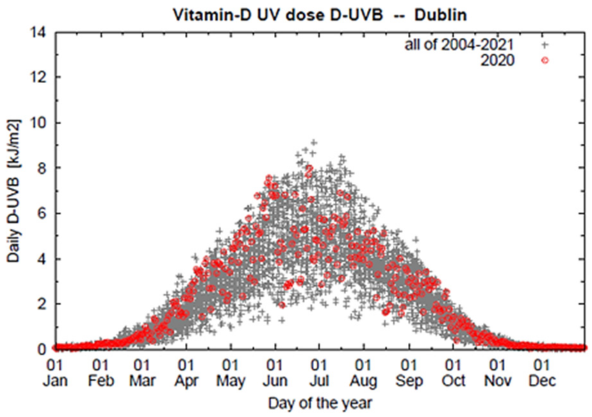

(C)

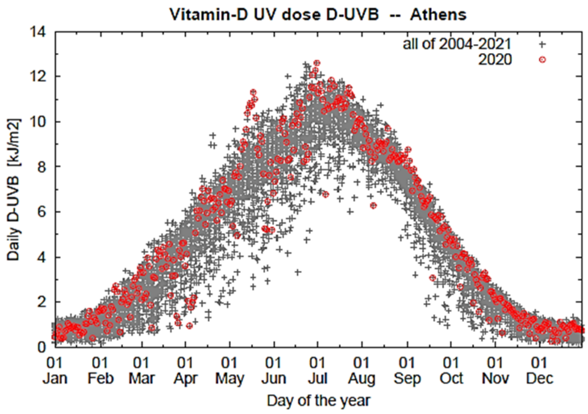

(D)

Figure S3. All measured diurnal D-UVB doses (2004-2021) for Dublin **(A)** and Athens **(B)** are shown, alongside the average D-UVB over the same period (in red). Diurnal D-UVB doses for 2020 only are shown in red, alongside all measured diurnal D-UVB values (2004-2021) for Dublin **(C)** and Athens **(D)**.
